# Can current post‐traumatic growth models capture the lived experience of life with a fluctuating chronic illness? Towards a new model

**DOI:** 10.1111/bjhp.70003

**Published:** 2025-06-30

**Authors:** Rachel Murphy, Belinda Harris

**Affiliations:** ^1^ University of Nottingham Nottingham UK

**Keywords:** chronic illness, complex trauma, trauma, traumatic chronic illness survival

## Abstract

**Introduction:**

More than 15 million people in the UK live with a chronic illness, and this figure is only due to increase (*Care and support for long term conditions*, 2024). Living with these illnesses can involve surviving ongoing trauma. Such trauma can lead to psychologically distressing emotions akin to post‐traumatic stress, where the self‐concept is threatened and disorganized (*Psychology and Psychotherapy: Theory, Research and Practice*, 2004, **77**, 101). Theories of post‐traumatic growth following such stress provide a useful lens for viewing traumatic events (*Rehabilitation Psychology*, 2014, **59**, 10). However, an alternative view may be needed when considering the trauma experienced by those living with chronic, fluctuating illnesses. This paper highlights the psychological impact of life with a chronic illness through an examination of women's experiences of living with inflammatory bowel disease. It examines the impact of this condition on their sense of self, including experiences of growth.

**Method:**

Semi‐structured interviews were conducted with 16 women within the UK, and the resulting co‐created data were analysed using the Stevick–Colaizzi–Keen method (*Phenomenological research methods*, 1994).

**Results:**

Four key interconnecting themes were identified: wearing the straitjacket of illness, which leads to psychologically difficult emotions, which are successfully managed, or not, through flexibility of self, which impacts the path to navigating a way through. A model of traumatic chronic illness survival emerged, which challenges counsellors and clinical psychologists to reconsider their preconceptions of those living with a chronic, retraumatising illness.

**Conclusion:**

Rather than holding an expectation of ‘growth’ due to illness, this model celebrates the multitude of ways people survive in the face of such devastating conditions.

## INTRODUCTION

It is estimated that between 30% and 40% of the global population lives with a gastrointestinal condition (GIC) (Sperber et al., 2021; Wang et al., 2023). Recent evidence suggests that inflammatory gastrointestinal conditions, collectively identified as inflammatory bowel disease (IBD), are becoming more prevalent globally, particularly in South Asian countries (Lin et al., 2024). A similar or higher incidence and prevalence of IBD has been reported in the UK (Freeman et al., 2021). Whilst incidence rates for gastrointestinal conditions tend to be evenly distributed between men and women, Severs et al. (2018) reported higher incidence rates of IBD in women than men, ranging from 1:1.2 (IBD) to 1:15 (Crohn's).

Common manifestations of IBD are the incurable diseases of the digestive system, ulcerative colitis (UC) and Crohn's disease (CD). IBD diagnosis indicates structural damage to the bowel caused by inflammation, with symptoms that include abdominal pain, diarrhoea and mucus, incontinence, fatigue, mouth ulcers, anaemia, and loss of weight and appetite (Crohn's and Colitis UK, 2019; Kaplan, 2018). IBD is also associated with extra‐intestinal manifestations that affect the eyes, skin, joints, bones, liver, and kidneys (Bernstein et al., 2019; Byrne et al., 2017; Greuter & Vavricka, 2019).

Often unpredictable, symptoms can range from chronically active to periods of remission. The severity of symptoms can change hourly, increasing the difficulty of managing such a condition. Whilst medication can control symptoms, up to 80% of people with CD undergo surgery (Crohn's and Colitis UK, 2018). This surgery can involve a bowel resection or the complete removal of the bowel and the introduction of a stoma. Whilst not life‐threatening, the symptoms and unpredictability of IBD, together with adverse side effects from some treatments, can significantly impact Quality of Life (Yeshi et al., 2020), including psychological, physical, sexual, and social functions (Byrne et al., 2017; Lenti et al., 2020).

The deleterious interrelationship of chronic illness and stress (Agorastos & Chrousos, 2022) is well documented. Rates of psychological distress (depression and anxiety) in patients with IBD are 20% higher than in the general population (Moulton et al., 2019). It is argued that the progression of IBD may be informed by and contribute to the environmental and psychological stress of those affected (Fairbrass et al., 2022). A recent systematic review identified stress both as a precursor to IBD diagnosis and to subsequent IBD exacerbation (Black et al., 2022); further, environmental factors contribute to a positive association between high levels of stress and relapse (Martin et al., 2015). It is also recognized that IBD diagnosis and treatments can elevate stress levels with potential consequences for the disease course (Sun et al., 2019).

When IBD strikes, previous identities associated with being unaware of, or unselfconscious about one's body, are challenged (Benner, 1994; Charmaz, 1983). This new IBD identity has been described as that of ‘other’; of not being integrated into a coherent view of self. Further, self‐confidence and sense of self are now associated with a perceived alignment between self‐perception and societal views of what constitutes a beautiful body (Moss & Dyck, 2002).

In a society where power is distributed in relation to “ability, age, citizenship, class, ethnicity, gender, health status, nationality, race, sex, sexuality, and other sets of relations we have yet to name” (Moss & Dyck, 2002, p.53), people with IBD are required to navigate their physical symptoms in public spaces, knowing their symptoms contravene societal norms of acceptable behaviour (Sajadinejad et al., 2012).

Additionally, living with unpredictable symptoms of such a fluctuating chronic illness can be traumatic (Glynn & Knowles, 2023; PURC‐Stephenson, 2014). There are times when illness is at the forefront of life, consuming all energy, and other times when wellness is figural (Paterson, 2001). Such fluctuations in illness status make acceptance difficult for the chronically ill; they live in “the dual kingdoms of the well and the sick” (Donnelly, 1993, p. 23).

In psychological terms, trauma‐related/induced feelings emanate from the process of adapting to a traumatic event, rather than the event itself (Williams, 2006). When seen through a person‐centred lens, such processing can highlight a disconnect between the ideal and real self (Rogers, 1951). Therefore, previous self‐concepts (ideal self/embodied self) are challenged. It is widely accepted that the traumatic experience of living with and adapting to IBD can contribute to post‐traumatic stress (PTS) and post‐traumatic stress disorder (PTSD).

According to ICD‐11 (2022), PTSD follows exposure to an extremely threatening or horrific external event, or a series of events. In common with DSM‐V, three core symptoms are described: intrusive thoughts, feelings or memories or re‐experiencing of the event(s); avoidance of thoughts, feelings or memories of people and situations associated with the event(s); hyperarousal and reactivity to a sense of current threat, including irritability, hypervigilance, startle response and sleep problems. A recent systematic review indicated that 31% of people with IBD meet the criteria for PTSD (Glynn & Knowles, 2023).

Although not life‐threatening, IBD is more akin to the complex trauma repeatedly experienced by abused or neglected children (Cámara et al., 2011; Johnson et al., 2012; Taft et al., 2021), with potential consequences for the development of personality (Dorahy et al., 2009). In contrast to DSM‐V, which does not recognize complex trauma as a separate condition, the ICD‐11 definition includes three additional trauma symptoms associated with ‘disturbances of the self‐organization’: problems in affect regulation; beliefs about self as defeated or diminished accompanied by feelings of shame, guilt, or failure related to the event(s); and difficulties in relating to and feeling close to others.

The literature on chronic illness reveals some significant conceptual differences between the DSM‐V definition of medical trauma (catastrophic external medical event(s) in the past e.g. surgical or treatment experiences that meet specific traumata), and definitions which also recognize the ongoing, repetitive somatic experiences of people living with cancer (Springer et al., 2023), HIV (Rzeszutek et al., 2019), heart disease (Edmondson, 2014) IBD (Sun et al., 2019).

The DSM‐V definition of medical trauma marginalizes PTSD in favour of ‘adjustment’ to critical life events (AjD). However, recent studies highlight conceptual confusion, when patients' symptoms meet the criteria for PTSD, and yet the prevalence of AjD remains relatively low, and the diagnosis of DSM‐V medical trauma is negative (Springer et al., 2023).

From a person‐centred theoretical perspective, Joseph (2004) described PTS as the self‐concept remaining threatened or disorganized whilst engaged in the process of understanding and making meaning about event(s) (Joseph, 2004). However, it is also argued that trauma experiences can precipitate the adjustment of one's world view and self‐concept, thereby reducing the distance between the ideal and real self (Joseph & Linley, 2005) and contributing to post‐traumatic growth.

Post‐traumatic growth (PTG), a concept first coined by Tedeschi and Calhoun (1996), is defined as “the potential of a dynamic system to adapt to adverse events, while hereby expanding its previous resources” (Mangelsdorf et al., 2019, p. 2). The organismic valuing theory of growth posits that trauma response is influenced by four factors: the extent to which (a) the traumatic event challenges pre‐existing world views; (b) organismic valuing has been encouraged by the social environment; (c) one's actualising tendency is tuned into and actioned; and (d) the current social environment encourages growth (Joseph et al., 2008; Joseph & Linley, 2005). These four factors influence whether the trauma experience is assimilated into the current world view or whether a new world view is developed to accommodate the traumatic event.

PTG, however, is a contested term. Studies of PTG, in people with breast cancer (Hefferon, 2012) and HIV (Rzeszutek et al., 2019), indicate that the relationship between PTG and health‐related well‐being is more nuanced and complex than the model suggests. It is argued that an individual's perceptions of their somatic symptoms are overlooked, and that psychosocial variables have a pivotal influence on an individual's health journey experience. Longitudinal data are needed to consider the relationship between the onset of illness and the development of PTG over time.

Critique from within the person‐centred community highlights the risk that individuals may be blamed for not attaining PTG (Lee, 2017). This critique is equally valid for the DSM‐V focus on ‘Adjustment Disorder’ (AjD) in medical situations. The authors have concerns about the usefulness and value of both AjD and PTG when considering the lived experience of people with complex, ongoing medical trauma, like IBD.

Systematic reviews have provided evidence that Quality of Life (Jones et al., 2019; Knowles, Graff, et al., 2018; Knowles, Keefer, et al., 2018) and psychological stress are negatively affected by IBD (Mikocka‐Walus et al., 2016). More recently, qualitative research has focused on the lived experiences of adults with IBD in the U.S. (Pothemont et al., 2021), Canada (Nistor et al., 2023; Popov et al., 2021), and Australia (Glynn & Knowles, 2023). These studies highlight the role of trauma at all stages of the illness journey and argue that the quality of care and treatment currently offered does little to recognize or mitigate its effects.

Women are disproportionately affected by IBD diagnosis, with a 20–30% higher risk of developing CD, and increased severity of symptoms, including sexual difficulties (Trachter et al., 2002). Recent studies undertaken in North America and Italy provide evidence that women experience lower quality of life, more psychological distress and less sexual activity than their male counterparts (Lungaro et al., 2023). Little is known about the lived experience of women with IBD in the UK. This doctoral research aimed to give voice to women with inflammatory bowel disease and to examine how the experience of IBD impacts a woman's sense of self.

## METHOD

An Interpretive Phenomenological Aproach (IPA) was chosen to explore the lived experience of women with IBD in the UK (Smith, 2011; Smith et al., 2009; Smith & Nizza, 2022), within a broader social, cultural and theoretical context (Larkin et al., 2006; Pietkiewicz & Smith, 2014), a key objective of this research. Phenomenological inquiry seeks to describe the whole person as a subject within their lived world, including their social and cultural influences. IPA is a qualitative approach which enables participants' stories to be told through the interpretive ability of the researcher. Smith & Osborn (2003, p. 51) use the term ‘dual process’ to describe the active process of researcher and participant co‐creating meaning together. In line with Husserl's principle of ‘epoche’ (Husserl, 1983), the IPA researcher approaches the phenomenon under investigation with an open mind and brackets off any existing knowledge or ideas until interpreting the data.

Research participants were recruited (Table 1) and provided with a participant information sheet and a consent form. Following brief communications with 21 volunteers to check they met the inclusion criterion of a formal diagnosis of IBD, semi‐structured interviews were conducted.

**TABLE 1:** Research participants.

Participant	Participants included in the research					
	Age	Self‐identified ethnicity	Recruitment method	Interview date	Interview location	Interview length (mins)
Mia	23	White British	CCUK website	14/06/19	University room	80
Wendy	54	White British	CCUK local meeting	20/06/19	Home	100
Michelle	64	White British	Facebook	29/07/19	Home	80
Suzie	41	White British	Facebook	01/08/19	Home	88
Sarah	44	White British	Facebook	09/09/19	Home	63
Higgler	72	White British	Facebook	12/09/19	Home	61
Jenny	27	White British	CCUK website	17/09/19	Meeting room at work	63
Claire	34	White British	Facebook	18/09/19	Café chosen by Claire	71
Sharon	32	White British	Facebook	14/10/19	Home	103
Kate	32	White British	CCUK website	24/10/19;and 29/01/20	University room and home	80
Sally	37	White British	CCUK	18/11/19	Home	90
Chloe	33	White British	CCUK website	20/11/19	Room in the community centre	74
Elsie	27	White British	CCUK website	30/11/19	Room in the community centre	96
Hannah	25	White British	CCUK website	01/12/19	Home	110
Katy	20	White British	CCUK website	04/12/19	University room	101
Ellie	47	White British	CCUK website	09/12/19	Home	104

Participants not included in the research	
Research number and recruitment method	Reason for non‐inclusion
07/Facebook	Agreement received and interview arranged. However, the interview was cancelled due to poor health, and no further contact was made, despite prompting
08/Facebook	Participant information sent, but no reply, despite prompting
14/Facebook	The potential participant was undiagnosed and therefore fell outside the research criteria. Details of the CCUK website were sent to the signpost support
18/CCUK website	Interview date arranged, but no location specified. No further communication despite prompting
20/CCUK website	Facetime call arranged, but the call was never made by 20. No further communication despite prompting
21/CCUK website	Interview date arranged, but no location specified. No further communication despite prompting
24/CCUK website	Participant information sent, but no reply received despite prompting

**FIGURE 1:**
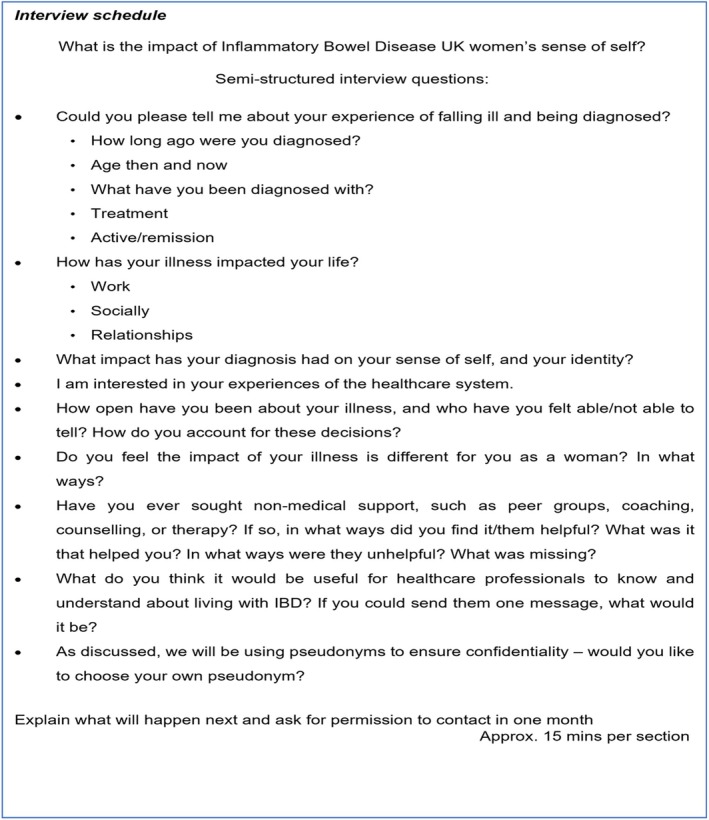
Interview schedule.

The interview schedule was comprised of open questions to elicit each participant's lived experience, rather than to address any predetermined criteria (Figure 1).

**FIGURE 2:**
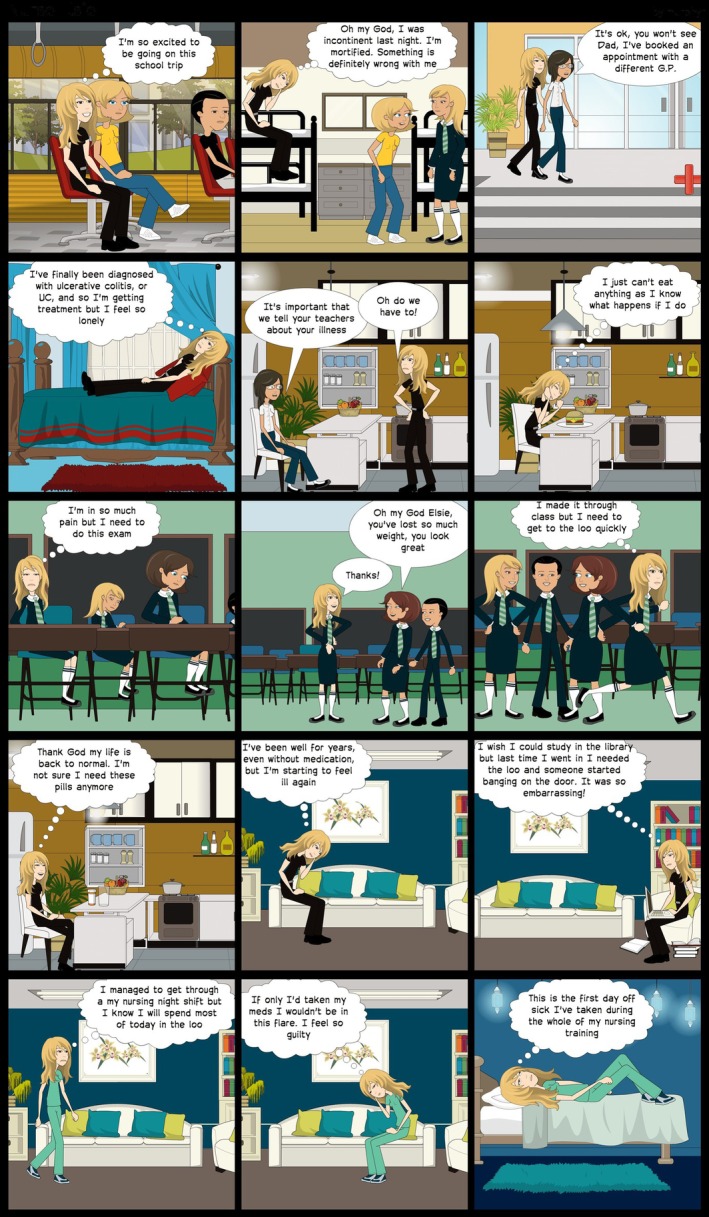
Section of Elsie's cartoon.

For accuracy, interviews were recorded, transcribed, and the transcriptions were sent to each participant for review, with an invitation to correct, edit, or add anything they had forgotten to say at the time. Before and immediately following each interview, the researcher (first author) made a reflexive journal entry, and after the participant's transcript had been approved and returned, they listened to the interview recordings several times, reread the transcripts and created a cartoon story to send to each participant (Figure 2) along with a summary of key themes from the interview.

Together, the summary and cartoon story provided evidence of deep listening and a concerted attempt to portray each person as a whole, rounded individual with their daily struggles and triumphs; this mitigated any risk of participants feeling objectified or divorced from their psychosocial reality (Rogers, 1951). The creation of each cartoon enabled an additional level of immersion in the research transcripts and provided a further opportunity for participants to give feedback on whether the thematic analysis resonated with and reflected their own understanding of their situation.

All participants approved the transcript of their interview, their cartoon and the researcher's summary of their individual themes. Drawing on the work of Hadfield and Haw (2001) the cartoons provided an authoritative voice as they were authentic and representative, particularly as the participants were authors, editors and final approvers of their own story. The gift of a bespoke cartoon for personal use was universally appreciated.

## ANALYSIS

The research data was co‐generated with participants through semi‐structured interviews. Supervision was essential to ensure vigilance regarding ethical and professional boundaries. The researcher drew on their background as a counsellor to listen deeply to words and silences (Charmaz, 2002). Counselling skills were supportive of relationship building with participants, whose traumatic stress had not been fully heard by family or medical professionals (Glynn & Knowles, 2023; Nistor et al., 2023; Popov et al., 2021). The chronically ill are acutely aware of the potential pain their stories may cause significant others and the price they may have to pay for having a voice. The researcher's professional identity and their own diagnosis of IBD (CD) were vital in gaining the trust of participants. Several reported a sense of relief that they might discuss the (elsewhere) undiscussable and valued being heard by a non‐judgmental ‘other’, intent on understanding their experiences.

**FIGURE 3:**
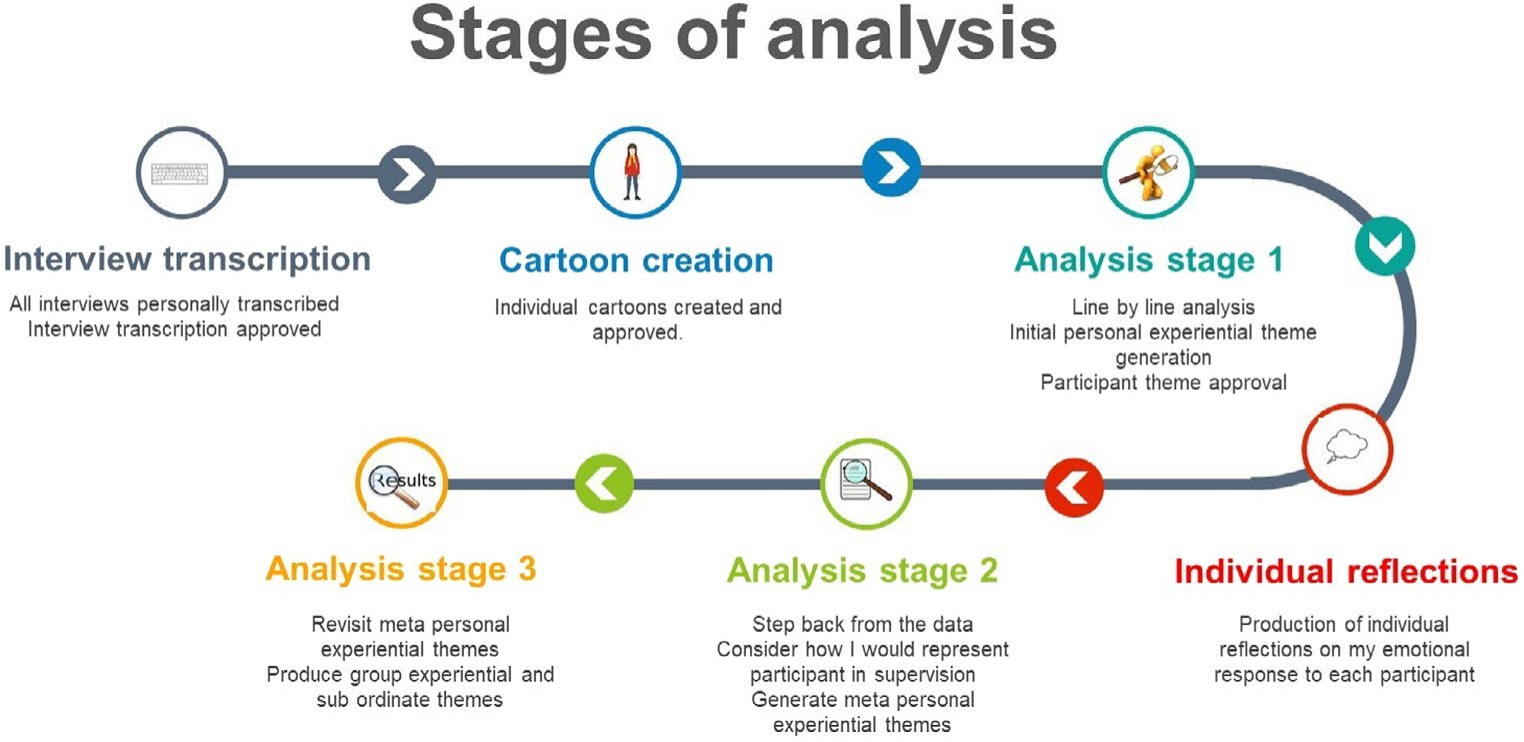
Stages of analysis.

The research analysis followed the modified Stevick–Colaizzi–Keen protocol for phenomenological analysis (Moustakas, 1994). This allowed for initial immersive analysis of each transcript individually. The analysis process then followed an ongoing hermeneutic cycle of moving back and forth within the text, shifting from a position of immersion within a case study to one of pan‐study theme consideration (Figure 3).

The second author, a senior qualitative researcher, reviewed and evaluated a sample of the transcripts, cartoons, individual thematic analyses and feedback from participants in regular supervisory meetings.

In line with Braun and Clarke's (2021) approach to reflexive thematic analysis, careful attention was paid to data saturation at every stage of the data collection phase to determine when no new information (themes or codes) was being obtained.

## ETHICAL CONSIDERATIONS

Ethical approval was gained from the University of Nottingham School of Education Ethics Committee, and rigorous dialogue with the research supervisors served to clarify the role of the researcher in this context and safeguard participants from any potential harm as a result of sharing their experiences. Participants were aware from the outset that the researcher was a PhD student and had been diagnosed with CD. The ‘process consent’ model was followed: consent was an ongoing process, re‐established and, where necessary, renegotiated throughout every stage of the research process (Etherington, 2007). In addition to written informed consent before enrollment in the study, participants provided verbal or written consent prior to the interview, upon approval of the interview transcripts, at a one‐month post‐interview check‐in meeting, and on approval of their themed narrative.

Due to the sensitive nature of this research, the interviews were conducted using a person‐centred approach, founded on facilitation of a safe environment, and characterized by undivided attention and unconditional respect for whatever issues they chose to share (Elmir et al., 2011; Rogers, 1951). An ethic and duty of care were figural throughout the process. Following each interview, participants were offered information about local counselling support, as well as details of the Crohn's and Colitis UK helpline. Three participants accessed counselling following their interview.

Close attention to ethical issues in supervision was particularly important given the first author's own IBD experience. The research process demanded flexibility of movement along a continuum from outsider researcher, and non‐judgmental, empathic counsellor, to insider with IBD, a challenge which became particularly acute during the pandemic (Murphy et al., 2022).

Observance of research ethics went beyond adherence to codes, frameworks and ‘dutiful ethics’ to embrace the researcher's principles, including non‐maleficence, beneficence, autonomy, justice, and fidelity (Etherington, 2007). Consideration was given to procedural ethics, following relevant codes of conduct, and to ethics in practice, the “day‐to‐day ethical issues that arise in the doing of research,” where unforeseen “ethically important moments” may arise (Guillemin & Gillam, 2004, p. 264).

Developing principled and nuanced reflexivity was an essential strategy for managing the tripartite relationship between participants, the research process, and IBD, and this was a consistent focus in supervision. Equally, identifying the ‘reflexive echoes’ of the researcher's own experience featured highly throughout the process, due to the sensitive and traumatic phenomena under investigation (Goldspink & Engward, 2019). The researcher was encouraged to take short periods of time away from the research to focus on their own well‐being; particularly to attend to personal material being triggered by the work and influencing their own sense of self. However, these periods of reflection provided a seam of deep, rich insight and interpretation, and were invariably followed by intense periods of writing.

## RESULTS

**FIGURE 4:**
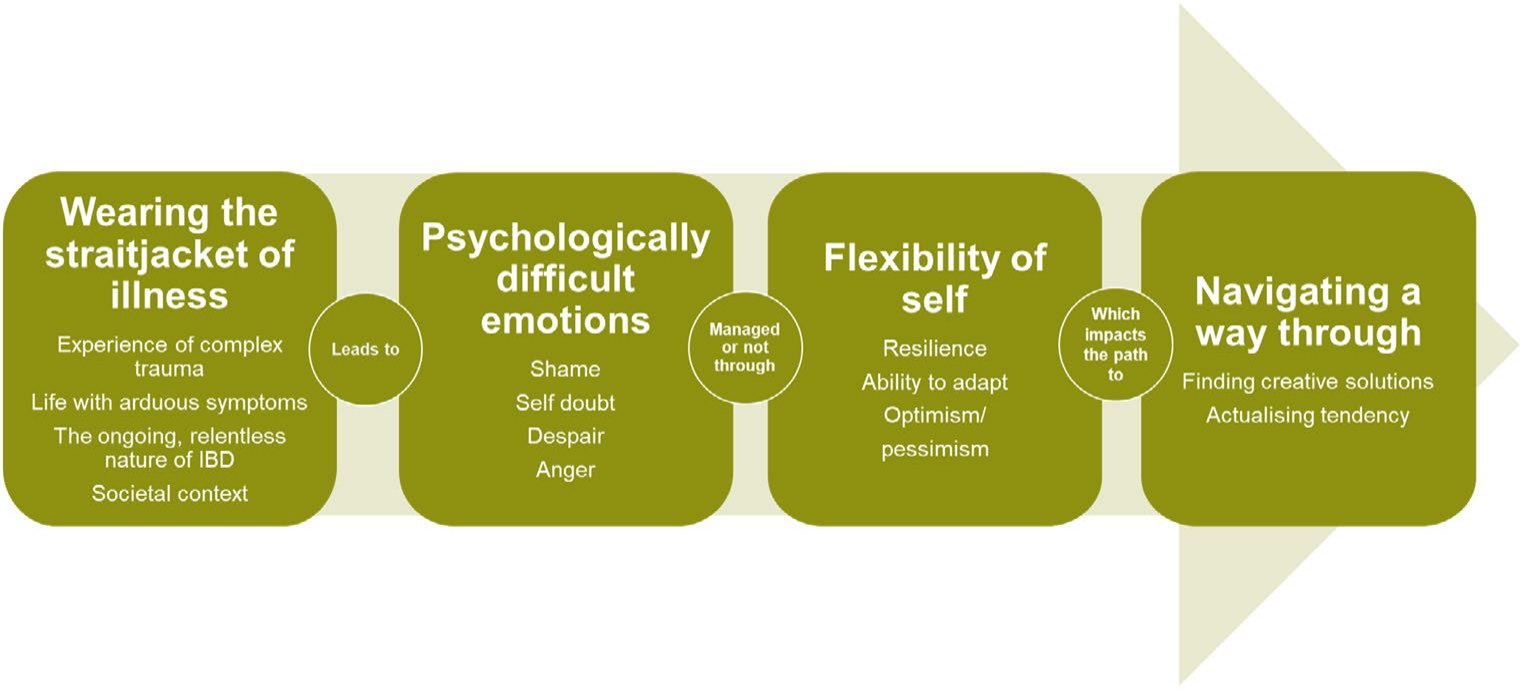
Group experiential themes.

The research data analysis resulted in four interconnected group experiential themes, which form an overarching narrative depicted in Figure 4.

### Theme one: Wearing the straightjacket of illness

Participants reported their illness experience as restrictive, akin to wearing a straitjacket. This jacket tightened or loosened in line with their position on the flare‐remission continuum. The straitjacket metaphor was indicative of complex trauma: life with relentlessly arduous symptoms, experienced within the context of a health‐prizing society.

Complex trauma had been triggered by the initial symptoms and diagnosis, was then intensified by the persistently fluctuating, life‐restricting nature of IBD, and deepened by intense feelings of difference. The women articulated this trauma clearly:

I lost four and a half stone in about three weeks…(Kate)

It is not knowing how you're going to be at any particular time and the uncertainty, the unpredictability… when it's bad, it's bad and I don't feel like I've got much grit and carry on then. (Higgler)

The sense of difference affected numerous areas of the participants' lives, especially where a stoma was present:

I think, like sexual, I think it affects sexuality. So, as a woman…it's really inconvenient in terms of like…when you're having sex, you've got this stoma bag flapping around. You can't be really spontaneous, you have to empty your stoma bag before even considering anything. (Sharon)

The numerous symptoms experienced were difficult to manage, with incontinence being the most emotionally and practically challenging:

I was on a night out once and I needed to go to the toilet there and then. Like I was desperate, and I had an accident, only a little one but it was enough to be, like devastating and I was like, I'm going home. That was horrible. (Sharon)

Frequent bowel movements associated with IBD were also debilitating, sapping women of energy and severely restricting their lives:

And…sometimes you'd rush to the loo and there's nothing much there…at the worse times I would say was about probably 12 times in half an hour or less. And as soon as I left the loo I had to go back again. (Higgler)

Besides frequent bowel movements, fatigue was cited as another incapacitating symptom and was experienced during a flare and in remission. It was often severe:

The thing I struggle with the most is fatigue. Like I feel I can deal with anything else that gets thrown at me, but it's the indescribable fatigue that you just can't get across to other people. (Sharon)

Society casts a shadow over all who are chronically ill. One manifestation of this was the experience of symptoms being missed, despite a palpable longing for understanding from others:

I tell my husband obviously. I don't think he really understands the problem, even though he's living with me. I don't think he really understands the problem, and perhaps he doesn't want to. (Higgler)

Participants reported that they often met a dismissive stance from healthcare professionals, which necessitated a concerted effort to feel heard and seen:

I don't want to have those conversations with doctors again where I have to fight for everything because I don't look sick enough. (Kate)

### Theme two: Psychologically difficult emotions

Difficult emotions, akin to PTS symptoms, had surfaced for many participants. For example, being unwell with such a difficult illness can lead a person to denial, defensiveness, minimization, and concealment. Such tactics may extend to the self, resulting in the experience of incongruence, a disconnect between the internal emotional landscape and the external self‐presentation. For the participants, these emotions fell broadly into four domains, namely shame, despair, self‐doubt, and anger.

The most endemic of these was shame, a cumulative, intense feeling of inadequacy and incompetence accompanied by fear of exposure. The shame experienced by the participants emanated from bowel symptoms which restricted their lives and led them to hide or minimize the illness experience. Feelings of self‐blame and being unlovable were common:

I feel like Monday to Wednesday… I can semi‐cope, Thursday isn't great. Friday is almost a write‐off for me working…I feel completely useless and so guilty about being useless or perceiving that I'm being useless. (Hannah)

It's obviously, because of the symptoms it's just like a bit of a stigmatising, embarrassing area to talk about…It's not something I do talk about (Ellie)

Or even, you know, going out and being a promiscuous teenager and thinking, oh my god. If I hadn't been doing that then, maybe I wouldn't have …Crohn's, I don't know. (Hannah)

I ended my seven‐year relationship in January because…I literally feel I don't have anything to give to a relationship. (Sally)

**FIGURE 5:**
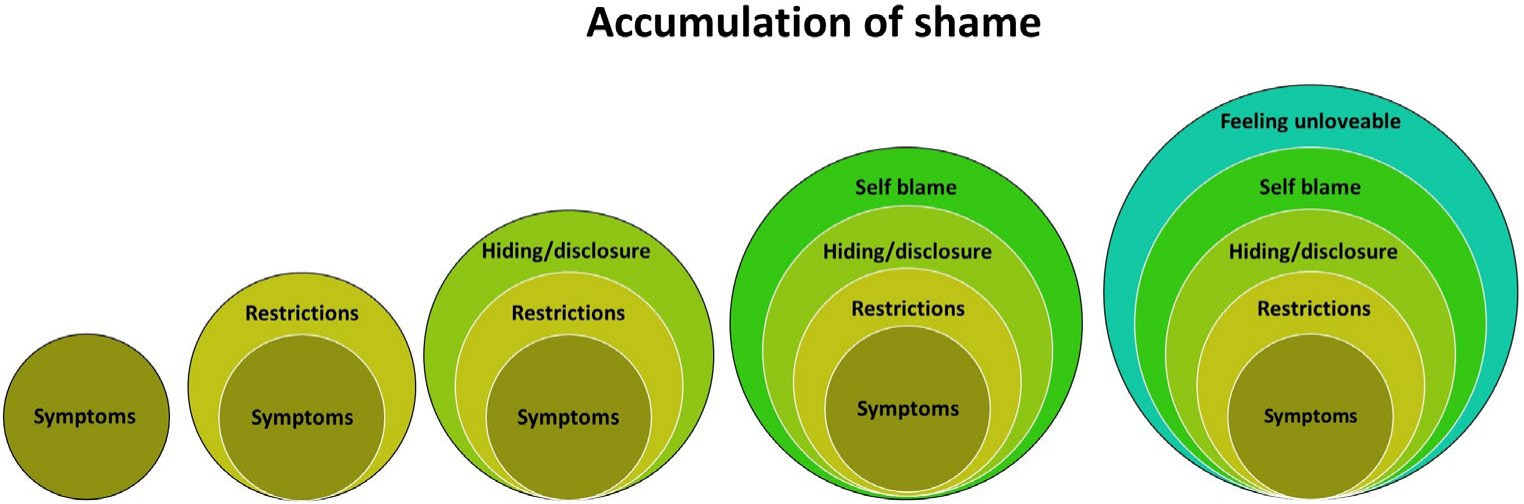
Accumulation of shame.

As shame accumulated it became a heavy burden to carry (Figure 5).

Retaining agency became difficult within the shame experience. Feelings of self‐doubt emerged, manifesting as constant internal self‐questioning. The doubting of the self was also imagined in the minds of others:

I don't want people to look at me and be, like ‘oh she's such a lazy person not pulling her weight’. But I also don't want them to look at me and say, ‘oh she keeps going on and moaning about her condition’. (Hannah)

Living with IBD often led the participants to feel despair or a loss of any real hope. This despair focused on the incurable nature of the illness; the depleted levels of resilience created by living with a chronic illness, curtailed lives and multi‐faceted, multi‐layered, deep‐seated fear:

But and then afterwards as, kind of, realisation hit more and more I became really, really upset with it. I was absolutely gutted by it, and I remember just being in bed crying thinking, oh my god, I've got this. I'm only 21. (Hannah)

Anger was also experienced by these women, although this was rarely expressed directly. Instead, it was communicated in more gentle, socially acceptable terms, such as frustration or sadness.

I don't want to be that person who's got Crohn's disease, so I didn't want it to be that that's what defines me as a human but now at this point, I feel like it bloody does. (Sally)

### Theme three: Flexibility of self

The analysis highlighted the importance of resilience, the ability to adapt, and optimism in achieving a level of self‐flexibility.

However, given the nature of the illness, such flexibility can feel difficult to obtain. The participants often showed resilience, both as a personality trait and as a process, in the face of their challenging disease. It should be noted that these types of resilience relate to surviving and recovering, not thriving.

Personality trait resilience was often displayed by the participants through pragmatism:

I can't really fight it. There's not really anything that I can do. I might as well just get on with it and learn more about the disease… (Mia)

The interconnection between inherent resilient personality traits and process resilience was also in play, with each one influencing the other. Personality traits of low resilience negatively impacted the progress of process resilience:

I would say I was always probably fairly anxious… I think that, maybe having a tendency to be anxious anyway…I am struggling…I'm finding it, you know, stressful or anxious or, and I've used phrases like, ‘I'm really struggling to cope with this’, quite a lot…(Suzie)

The practical ability to adapt to a new illness reality was facilitated through psychological adaptation, lessening the debilitating impact of IBD:

I think the fact that I've, sort of, come to terms with it now and I know that I can still get everything I want done…without it having too much of an impact on my health. (Mia)

The participants displayed how living with IBD was challenging, and therefore, optimism could prove elusive. However, being able to feel optimistic showed a level of flexibility of a sense of self necessary to survive a socially unacceptable illness. Such optimism could mitigate against the fear of the future:

It's one of those, I know that I'm very self‐aware and I know that I know very much what I want to do and how I need to achieve it. (Mia)

Actively choosing to release any idea of control over IBD and accept what comes sometimes engendered a sense of optimism:

There's no rhyme or reason to it, so I think the best thing that I've learnt really is just to try and stay positive about it. (Claire)

The integration of illness into the sense of self involved a degree of acceptance:

I'm, like it's just a normal fact, like I have brown hair, I have Crohn's disease, I have this, I have that. (Mia)

### Theme four: Navigating a way through

As shown in Theme 3, the participants demonstrated a certain level of flexibility of self through resilience, adaptability, and optimism. This placed them in a position to find ways to navigate their illness. They found creative solutions and reaffirmed their actualising tendency, the capacity to develop capabilities that maintain or enhance the self. Tapping into this innate motivation appeared to enhance their movement towards potentiality. The ability to find a way to navigate to a new reality impacted their quality of life and ultimately their progression towards functioning as a whole person: someone open to experiences, who trusts their own organism and can live in the moment. The participants who were able to move in this direction had developed creative methods to navigate through their illness and reaffirm their actualising tendency:

The conversation [with others] is no, no longer focussed on, I have the disease. It's I have a disease, but I'm doing this. (Katy)

…that's when I had the whole thing removed…Oh I love it, it's so good…It [her stoma] is incredible. Mine's very nice, I like him. (Michelle)

Navigating a way through was also achieved through self‐care:

I probably make unconscious decisions to protect myself from a flare up every day. (Jenny)

However, navigating a way through IBD was shown to be challenging, with only half of the participants displaying some degree of movement in this area. Difficulty in managing symptoms, making comparisons with others, and societal pressures were some of the apparent barriers to finding a way to navigate the illness journey:

“I don't think I, I don't think I've probably still fully got my head round going from being someone who didn't have any chronic illness, or any serious health problems, to having two debilitating illnesses.” (Suzie)

“I ended up with depression because the pain was so…the pain was just, just, just like, if it had continued, I wouldn't be here…It wasn't liveable with.” (Suzie)

“…on Saturday, I felt quite unwell with abdo pain and it made me feel so guilty and upset for not doing chores/exercise/jobs etc. It feels like symptoms get in the way so much of accomplishing what others can do easily.” (Elsie)

Lack of psychological support could be considered another reason why navigating a way through appeared to be so difficult. Of the 16 participants, only one was offered psychological support to specifically help with living with IBD.

## DISCUSSION

Central to critiques of PTSD (cf heart disease, Edmondson, 2014) and PTG (cf. cancer, Springer et al., 2023) is the neglect of corporeal, somatically experienced trauma, focused on here‐and‐now pain, together with fear of further attacks and mortality. This study provides some evidence that participants were preoccupied with the physical effects of their condition and how these affected their immediate and longer‐term situations.

PTG is understood to have occurred when positive changes take place following trauma in the areas of relationships, self‐concept and life philosophy (Joseph et al., 2012; Linley & Joseph, 2004; Murphy & Joseph, 2013). A recent Chinese study tracked 16 people newly diagnosed with Crohn's for 1 year. PTG was reported, citing closer relationships with others, increased personal strength, changes in life philosophy and emerging new possibilities (Chen et al., 2024).

Our participants had lived with IBD for between 11 months and 18 years and experienced several relapses and remission cycles. The findings identified a potential route to living whole lives within the limitations of disease severity. However, it is important not to lose sight of the challenges involved, given the intense ongoing trauma experienced by the participants.

Although some participants found ways of accommodating their illness, it was not an overall positive experience, with no predictable timeline or anything to be grateful for. Only one participant attributed their illness to a positive change. This aligned with PURC‐Stephenson et al. (2015) results, where 73% of participants had reported a positive impact and 80% reported the disease had negatively impacted life, illustrating the nuanced, ambivalent relationship women living with IBD have with their illness.

**FIGURE 6:**
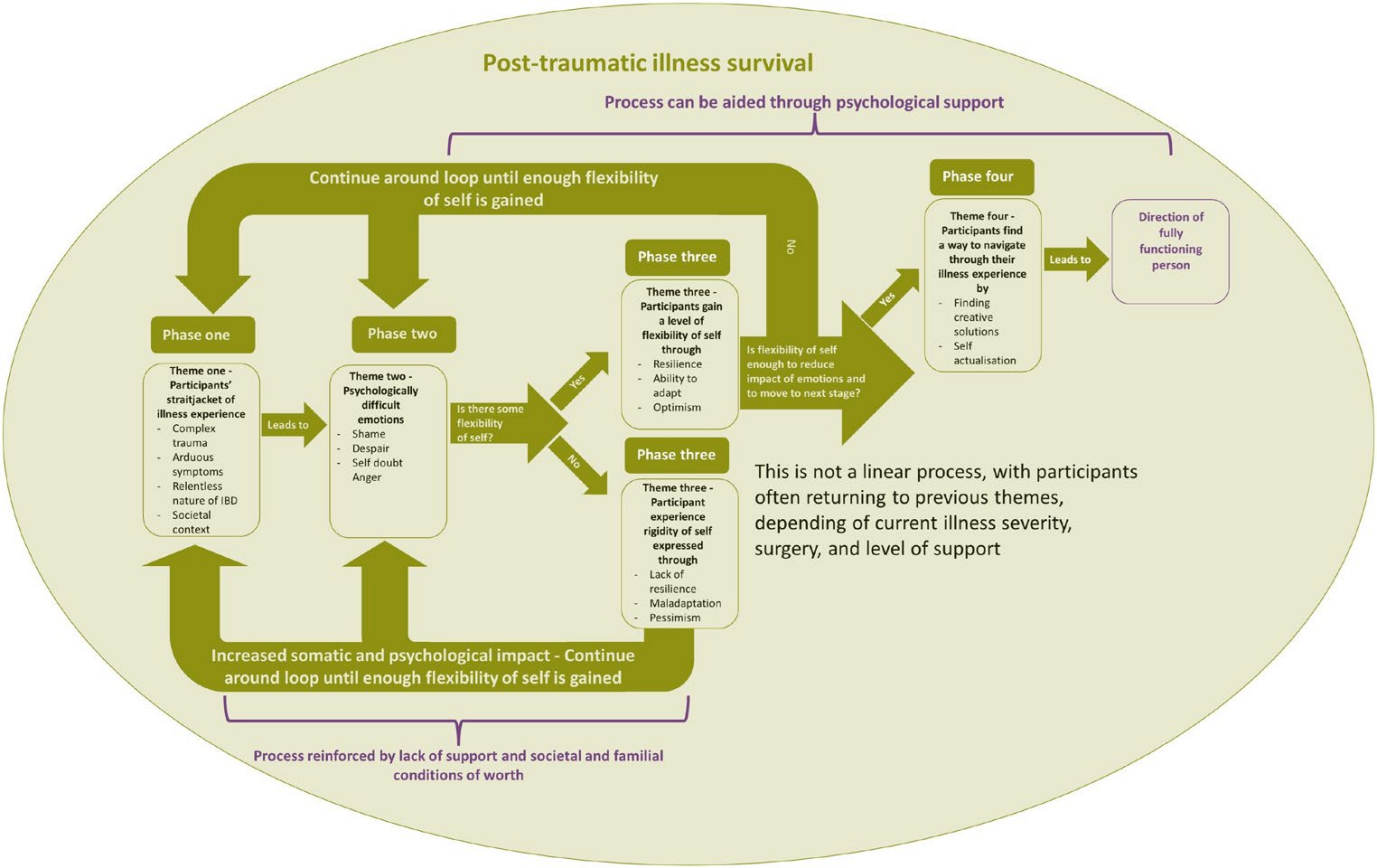
Model of traumatic chronic illness survival.

In line with research into life‐threatening and non‐life‐threatening chronic illnesses, this research challenges the PTG models by highlighting the struggle to maintain positive growth in the face of a negative, somatic, stigmatizing, and persistent traumatic disease. Whilst adjustment and PTG models are useful when considering one‐off medical trauma, an ongoing, retraumatising situation requires an alternative lens; one that reflects the cyclical nature of such experiences. A model of traumatic chronic illness survival (TCIS) was created (Figure 6).

This TCIS model does not accord with the view that suffering is a necessary path to growth; pain and anguish are the unfortunate effects of a devastating illness. TCIS prizes the ability to survive under such extreme circumstances. It illustrates the psychological burden of life with a fluctuating chronic illness and contextualizes the lives of the chronically ill in a way that supports both those living with illness and clinical professionals working with them. The vital role of empathetic support is recognized for managing life with such a debilitating condition.

This model provides structure to the often‐cyclical movement those with IBD undergo in their bid to make sense of their traumatic illness experience. Four non‐linear phases are navigated, with movement in both directions between phases, as trauma is reexperienced and processed. Research participants often found themselves at phase one after experiencing further traumatic somatic symptoms, despite having previously progressed through further phases. However, this research also indicated that prior progress through the later phases contributed to shorter progression to the next phase.

A much‐prized flexibility of self was often hard to achieve, especially when the disease experience was severe and traumatic; unhelpful societal and familial conditions of worth contributed, as did lack of support. Increased somatic and psychological impacts were experienced, and re‐traumatisation occurred, resulting in movement back to phase one.

Where a readjustment of the sense of self was possible, but incorporation of the illness experience was partial, a return to the earlier phases of the model occurred. This process continued until the sense of self was realigned, feelings of incongruence were reduced, and the impact of the resultant psychological emotions lessened. Such progress is particularly important for people with IBD, as difficult psychological emotions have been shown to increase disease activity (Farrokhyar et al., 2006; Kochar et al., 2018; Mardini et al., 2004; Maunder & Levenstein, 2008; Mittermaier et al., 1998).

A reduction in disease activity could potentially reduce the need for medical interventions, increase quality of life, and reduce the impact on the NHS. Additionally, achieving flexibility of the sense of self supported the progression to phase four of the model, where creative and self‐actualising solutions were developed. Progression through phases two, three, and four could be aided by effective psychological support, which is founded on understanding the real impact of a chronic illness, as contextualized within the model.

Reaching phase four of the model was unusual, with participants more often circulating around the first three phases. This was the reality for women living with IBD; managing to survive complex trauma rather than experiencing a sense of peace and growth. Therefore, this model aids empathetic understanding of the challenges faced by people living with chronic illness and mitigates against an expectation of psychological growth. Such an expectation may prevent deep psychological contact. Survival is enough, growth is a bonus.

This TSIC model has the potential to be translated across other chronic illnesses, providing a way to conceptualize the illness trauma experience and consider effective psychological interventions.

## LIMITATIONS

This study has merit in that it offers depth of insight into the social and emotional experience of women of all ages diagnosed with IBD. Cartoon stories encapsulate and honour participants' life experiences. However, the sample consisted predominantly of white, middle‐class women and is therefore not representative of the wider UK demographics. Participants had actively chosen to engage with the research process and therefore had sufficient awareness and confidence to articulate their experiences. Despite the checks and balances of supervision, the doctoral researcher with IBD was also the main data analyst, with implications for the interpretation of data. The findings could have been more nuanced had more data about each participant been collected.

Further studies are needed that include women from diverse racial and cultural backgrounds to see whether the themes identified are consistent or require further refinement. Longitudinal research could also explore changes in emotional states over time and trial the TCIS model to evaluate its relevance and utility for diverse cultures, healthcare professionals, people with IBD and other chronic illnesses.

## CONCLUSION

With an increasing number of people experiencing chronic illness in the UK (Nuffield Trust, 2024), logic suggests that the number of clients seeking psychological support for such an illness will also increase. Therefore, enhancing understanding of such lives is increasingly important.

Current models of medical trauma and PTG are useful when contextualizing one‐off trauma. However. the literature on chronic illness reveals significant conceptual differences between the DSM‐V definition of medical trauma (catastrophic external medical event(s) in the past e.g. surgical or treatment experiences that meet specific trauma), and definitions which also recognize the ongoing, fluctuating somatic experiences of people living with cancer (Springer et al., 2023), HIV (Rzeszutek et al., 2019) heart disease (Edmondson, 2014) and IBD (Sun et al., 2019). A more nuanced DSM‐V definition and symptomatology are needed to encompass more complex, chronic illnesses.

Healthcare professionals working with women living with IBD and other chronic illnesses would benefit from examination of their unconscious expectations of growth, or they may miss the outstanding achievement of traumatic chronic illness survival. Unexamined expectations can lead to silencing real experiences and instil a sense of failure in women who do not achieve PTG. This aligns with the research participant's feedback on being interviewed by someone who experienced similar struggles.

It is posited that traumatic chronic illness survival can include deep acceptance of life with a chronic illness and therefore a form of self‐actualization in practice. It enables women living with IBD to experience their symptoms and the resultant psychologically difficult emotions, and embark on the process of gaining more flexibility of self. They can, therefore, potentially establish a way to navigate through their illness and progress in the direction of a fully functioning person, without shame or feeling a failure during this long, complex journey. On each step of the way, they are surviving and that is not only sufficient, but awe‐inspiring.

## POSTSCRIPT

Unfortunately, the first author's health prevents them from working therapeutically with the TCIS model, and therefore, they offer TCIS to all professionals who work with the chronically ill, to trial the model and provide constructive feedback.

## AUTHOR CONTRIBUTIONS

**Rachel Murphy:** Conceptualization; investigation; writing – original draft; methodology; validation; visualization; writing – review and editing; formal analysis; project administration. **Belinda Harris:** Methodology; validation; writing – review and editing; supervision.

## CONFLICT OF INTEREST STATEMENT

There are no conflicts of interest.

## Data Availability

Data available on request due to privacy/ethical restrictions.

## References

[bjhp70003-bib-0001] Agorastos, A. , & Chrousos, G. P. (2022). The neuroendocrinology of stress: The stress‐related continuum of chronic disease development. Molecular Psychiatry, 27(1), 502–513. 34290370 10.1038/s41380-021-01224-9

[bjhp70003-bib-0002] Benner, P. E. (1994). Interpretive phenomenology: embodiment, caring, and ethics in health and illness. Calif, Sage Publications.

[bjhp70003-bib-0003] Bernstein, C. N. , Benchimol, E. I. , Bitton, A. , Murthy, S. K. , Nguyen, G. C. , Lee, K. , COOKE‐Lauder, J. , & Kaplan, G. G. (2019). The impact of inflammatory bowel disease in Canada 2018: Extra‐intestinal diseases in IBD. Journal of the Canadian association of Gastroenterology, 2(Supplement_1), S73–S80. 31294387 10.1093/jcag/gwy053 PMC6512250

[bjhp70003-bib-0004] Black, J. , Sweeney, L. , Yuan, Y. , Singh, H. , Norton, C. , & CZUBER‐Dochan, W. (2022). Systematic review: The role of psychological stress in inflammatory bowel disease. Alimentary Pharmacology & Therapeutics, 56(8), 1235–1249. 36082403 10.1111/apt.17202 PMC9825851

[bjhp70003-bib-0005] Braun, V. , & Clarke, V. (2021). One size fits all? What counts as quality practice in (reflexive) thematic analysis? Qualitative Research in Psychology, 18(3), 328–352.

[bjhp70003-bib-0006] Byrne, G. , Rosenfeld, G. , Leung, Y. , Qian, H. , Raudzus, J. , Nunez, C. , & Bressler, B. (2017). Prevalence of anxiety and depression in patients with inflammatory bowel disease. Canadian Journal of Gastroenterology and Hepatology, 2017, 6496727. 29181373 10.1155/2017/6496727 PMC5664260

[bjhp70003-bib-0007] Cámara, R. J. , Gander, M.‐L. , Begré, S. , Von Känel, R. , & Swiss Inflammatory Bowel Disease Cohort Study Group . (2011). Post‐traumatic stress in Crohn's disease and its association with disease activity. Frontline Gastroenterology, 2(1), 2–9. 24349679 10.1136/fg.2010.002733 PMC3854716

[bjhp70003-bib-0008] Charmaz, K. (1983). Loss of self: A fundamental form of suffering in the chronically ill. Sociology of Health & Illness, 5(2), 168–195. 10261981 10.1111/1467-9566.ep10491512

[bjhp70003-bib-0009] Charmaz, K. (2002). Stories and silences: Disclosures and self in chronic illness. Qualitative Inquiry, 8(3), 302–328.

[bjhp70003-bib-0010] Chen, L. , Zhou, Y. , & Liu, J. (2024). N12 the experience of post‐traumatic growth process in newly diagnosed Chinese patients with Crohn's disease: A longitudinal qualitative study. Journal of Crohn's and Colitis, 18(Supplement_1), i2212–i2213.

[bjhp70003-bib-0011] Crohn's and Colitis UK . (2018). 75% of people with Crohn's or Colitis have had an accident in public because they couldn't get to the toilet in time. https://www.facebook.com/crohnsandcolitisuk/photos/a.212445248781539/2876509555708415/?Type=3&theater.

[bjhp70003-bib-0012] Crohn's and Colitis UK . (2019). About inflammatory bowel disease. https://www.crohnsandcolitis.org.uk/about‐inflammatory‐bowel‐disease

[bjhp70003-bib-0013] Donnelly, G. F. (1993). Chronicity: Concept and reality. Holistic Nursing Practice, 8(1), 1–7. 7693738 10.1097/00004650-199310000-00003

[bjhp70003-bib-0014] Dorahy, M. J. , Corry, M. , Shannon, M. , Macsherry, A. , Hamilton, G. , Mcrobert, G. , Elder, R. , & Hanna, D. (2009). Complex Ptsd, interpersonal trauma and relational consequences: Findings from a treatment‐receiving northern Irish sample. Journal of Affective Disorders, 112(1–3), 71–80. 18511130 10.1016/j.jad.2008.04.003

[bjhp70003-bib-0015] Edmondson, D. (2014). An enduring somatic threat model of posttraumatic stress disorder due to acute life‐threatening medical events. Social and Personality Psychology Compass, 8(3), 118–134. 24920956 10.1111/spc3.12089 PMC4048720

[bjhp70003-bib-0016] Elmir, R. , Schmied, V. , Jackson, D. , & Wilkes, L. (2011). Interviewing people about potentially sensitive topics. Nurse Researcher, 19(1), 12–16. 22128582 10.7748/nr2011.10.19.1.12.c8766

[bjhp70003-bib-0017] Etherington, K. (2007). Ethical research in reflexive relationships. Qualitative Inquiry, 13(5), 599–616.

[bjhp70003-bib-0018] Fairbrass, K. M. , Gracie, D. J. , & Ford, A. C. (2022). Relative contribution of disease activity and psychological health to prognosis of inflammatory bowel disease during 6.5 years of longitudinal follow‐up. Gastroenterology, 163(1), 190–203. e5. 35339461 10.1053/j.gastro.2022.03.014

[bjhp70003-bib-0019] Farrokhyar, F. , Marshall, J. K. , Easterbrook, B. , & Irvine, E. J. (2006). Functional gastrointestinal disorders and mood disorders in patients with inactive inflammatory bowel disease: Prevalence and impact on health. Inflammatory Bowel Diseases, 12(1), 38–46. 16374257 10.1097/01.mib.0000195391.49762.89

[bjhp70003-bib-0020] Freeman, K. , Ryan, R. , Parsons, N. , TAYLOR‐Phillips, S. , Willis, B. H. , & Clarke, A. (2021). The incidence and prevalence of inflammatory bowel disease in UK primary care: A retrospective cohort study of the IQVIA medical research database. BMC Gastroenterology, 21, 1–7. 33771127 10.1186/s12876-021-01716-6 PMC8004426

[bjhp70003-bib-0021] Glynn, H. , & Knowles, S. R. (2023). A phenomenological investigation of trauma in 18 adults living with inflammatory bowel disease. Clinical Nursing Research, 32(1), 159–170. 35156407 10.1177/10547738221075649

[bjhp70003-bib-0022] Goldspink, S. , & Engward, H. (2019). Booming clangs and whispering ghosts: Attending to the reflexive echoes in IPA research. Qualitative Research in Psychology, 16(2), 291–304.

[bjhp70003-bib-0023] Greuter, T. , & Vavricka, S. R. (2019). Extraintestinal manifestations in inflammatory bowel disease–epidemiology, genetics, and pathogenesis. Expert Review of Gastroenterology & Hepatology, 13(4), 307–317. 30791773 10.1080/17474124.2019.1574569

[bjhp70003-bib-0024] Guillemin, M. , & Gillam, L. (2004). Ethics, reflexivity, and ‘ethically important moments’ in research. Qualitative Inquiry, 10(2), 261–280.

[bjhp70003-bib-0025] Hadfield, M. , & Haw, K. (2001). ‘Voice’, young people and action research. Educational Action Research, 9(3), 485–502.

[bjhp70003-bib-0026] Hefferon, K. (2012). Bringing back the body into positive psychology: The theory of corporeal posttraumatic growth in breast cancer survivorship. Psychology, 3(12A), 1238–1242.

[bjhp70003-bib-0027] Husserl, E. (1983). Ideas pertaining to a pure phenomenology and to a phenomenological philosophy: First book: General introduction to a pure phenomenology. Springer Science & Business Media.

[bjhp70003-bib-0028] Johnson, R. , Haigh, R. , & Benge, M. (2012). Complex trauma and its effects: Perspectives on creating an environment for recovery. Pavilion Publishing.

[bjhp70003-bib-0029] Jones, J. L. , Nguyen, G. C. , Benchimol, E. I. , Bernstein, C. N. , Bitton, A. , Kaplan, G. G. , Murthy, S. K. , Lee, K. , COOKE‐Lauder, J. , & Otley, A. R. (2019). The impact of inflammatory bowel disease in Canada 2018: Quality of life. Journal of the Canadian Association of Gastroenterology, 2(Supplement_1), S42–S48. 31294384 10.1093/jcag/gwy048 PMC6512247

[bjhp70003-bib-0030] Joseph, S. (2004). Client‐centred therapy, post‐traumatic stress disorder and post‐traumatic growth: Theoretical perspectives and practical implications. Psychology and Psychotherapy: Theory, Research and Practice, 77(1), 101–119. 10.1348/147608304322874281 15025907

[bjhp70003-bib-0031] Joseph, S. , & Linley, P. A. (2005). Positive adjustment to threatening events: An organismic valuing theory of growth through adversity. Review of General Psychology, 9(3), 262–280.

[bjhp70003-bib-0032] Joseph, S. , Linley, P. A. , Joseph, S. , & Linley, P. (2008). Positive psychological perspectives on posttraumatic stress: An integrative psychosocial framework. In Trauma, recovery, and growth: positive psychological perspectives on posttraumatic stress (pp. 3–20). John Wiley & Sons.

[bjhp70003-bib-0033] Joseph, S. , Murphy, D. , & Regel, S. (2012). An affective–cognitive processing model of post‐traumatic growth. Clinical Psychology & Psychotherapy, 19(4), 316–325. 22610981 10.1002/cpp.1798

[bjhp70003-bib-0034] Kaplan, G. E. A. (2018). Impact of inflammatory bowel disease in Canada. Crohn's and Colitis Canada.

[bjhp70003-bib-0035] Knowles, S. R. , Graff, L. A. , Wilding, H. , Hewitt, C. , Keefer, L. , & MIKOCKA‐Walus, A. (2018). Quality of life in inflammatory bowel disease: A systematic review and meta‐analyses—Part I. Inflammatory Bowel Diseases, 24(4), 742–751. 29562277 10.1093/ibd/izx100

[bjhp70003-bib-0036] Knowles, S. R. , Keefer, L. , Wilding, H. , Hewitt, C. , Graff, L. A. , & MIKOCKA‐Walus, A. (2018). Quality of life in inflammatory bowel disease: A systematic review and meta‐analyses—Part II. Inflammatory Bowel Diseases, 24(5), 966–976. 29688466 10.1093/ibd/izy015

[bjhp70003-bib-0037] Kochar, B. , Barnes, E. L. , Long, M. D. , Cushing, K. C. , Galanko, J. , Martin, C. F. , Raffals, L. E. , & Sandler, R. S. (2018). Depression is associated with more aggressive inflammatory bowel disease. The American Journal of Gastroenterology, 113(1), 80–85. 29134965 10.1038/ajg.2017.423 PMC5962285

[bjhp70003-bib-0038] Larkin, M. , Watts, S. , & Clifton, E. (2006). Giving voice and making sense in interpretative phenomenological analysis. Qualitative Research in Psychology, 3(2), 102–120.

[bjhp70003-bib-0039] Lee, D. (2017). A person‐centred political critique of current discourses in post‐traumatic stress disorder and post‐traumatic growth. Psychotherapy and Politics International, 15, e1411.

[bjhp70003-bib-0040] Lenti, M. V. , Cococcia, S. , Ghorayeb, J. , DI Sabatino, A. , & Selinger, C. P. (2020). Stigmatisation and resilience in inflammatory bowel disease. Internal and Emergency Medicine, 15(2), 211–223. 31893346 10.1007/s11739-019-02268-0 PMC7054377

[bjhp70003-bib-0041] Lin, D. , Jin, Y. , Shao, X. , Xu, Y. , Ma, G. , Jiang, Y. , Xu, Y. , Jiang, Y. , & Hu, D. (2024). Global, regional, and national burden of inflammatory bowel disease, 1990–2021: Insights from the global burden of disease 2021. International Journal of Colorectal Disease, 39(1), 139. 39243331 10.1007/s00384-024-04711-x PMC11380638

[bjhp70003-bib-0042] Linley, P. A. , & Joseph, S. (2004). Positive change following trauma and adversity: A review. Journal of Traumatic Stress, 17(1), 11–21. 15027788 10.1023/B:JOTS.0000014671.27856.7e

[bjhp70003-bib-0043] Lungaro, L. , Costanzini, A. , Manza, F. , Barbalinardo, M. , Gentili, D. , Guarino, M. , Caputo, F. , Zoli, G. , DE Giorgio, R. , & Caio, G. (2023). Impact of female gender in inflammatory bowel diseases: A narrative review. Journal of Personalized Medicine, 13(2), 165. 36836400 10.3390/jpm13020165 PMC9958616

[bjhp70003-bib-0044] Mangelsdorf, J. , Eid, M. , & Luhmann, M. (2019). Does growth require suffering? A systematic review and meta‐analysis on genuine posttraumatic and postecstatic growth. Psychological Bulletin, 145(3), 302–338. 30589274 10.1037/bul0000173

[bjhp70003-bib-0045] Mardini, H. E. , Kip, K. E. , & Wilson, J. W. (2004). Crohn's disease: A two‐year prospective study of the association between psychological distress and disease activity. Digestive Diseases and Sciences, 49(3), 492. 15139504 10.1023/b:ddas.0000020509.23162.cc

[bjhp70003-bib-0046] Martin, T. D. , Chan, S. S. , & Hart, A. R. (2015). Environmental factors in the relapse and recurrence of inflammatory bowel disease: A review of the literature. Digestive Diseases and Sciences, 60, 1396–1405. 25407806 10.1007/s10620-014-3437-3

[bjhp70003-bib-0047] Maunder, R. G. , & Levenstein, S. (2008). The role of stress in the development and clinical course of inflammatory bowel disease: Epidemiological evidence. Current Molecular Medicine, 8(4), 247–252. 18537632 10.2174/156652408784533832

[bjhp70003-bib-0048] Mikocka‐Walus, A. , Knowles, S. R. , Keefer, L. , & Graff, L. (2016). Controversies revisited: A systematic review of the comorbidity of depression and anxiety with inflammatory bowel diseases. Inflammatory Bowel Diseases, 22(3), 752–762. 26841224 10.1097/MIB.0000000000000620

[bjhp70003-bib-0049] Mittermaier, C. , Beier, M. , Tillinger, W. , Gangl, A. , & Moser, G. (1998). Impact of depressive mood on relapse of patients with inflammatory bowel disease (IBD): A prospective one year follow‐up study. Gastroenterology, 114(4), A1040–A1041. 10.1097/01.psy.0000106907.24881.f2 14747641

[bjhp70003-bib-0050] Moss, P. , & Dyck, I. (2002). Women, body, illness: Space and identity in the everyday lives of women with chronic illness. Rowman & Littlefield Publishers Ltd.

[bjhp70003-bib-0051] Moulton, C. D. , Pavlidis, P. , Norton, C. , Norton, S. , Pariante, C. , Hayee, B. , & Powell, N. (2019). Depressive symptoms in inflammatory bowel disease: An extraintestinal manifestation of inflammation? Clinical and Experimental Immunology, 197(3), 308–318. 30762873 10.1111/cei.13276 PMC6693970

[bjhp70003-bib-0052] Moustakas, C. (1994). Phenomenological research methods. Sage publications.

[bjhp70003-bib-0053] Murphy, D. , & Joseph, S. (2013). Facilitating posttraumatic growth through relational depth. In Relational depth: new perspectives and developments (Vol. 90, pp. 89–148). Palgrave Macmillan Ltd.

[bjhp70003-bib-0054] Murphy, R. , Harris, B. , & Wakelin, K. (2022). Riding a rollercoaster in a hurricane – Researching my own chronic illness. Qualitative Research Journal, 22(2), 248–260.

[bjhp70003-bib-0055] Nistor, O.‐I. , Wilson, R. , Tripp, D. , & CAMARGO‐Plazas, P. (2023). The lived experiences of discomfort in women with Crohn's disease: A hermeneutic phenomenological study. Gastrointestinal Nursing, 21(1), 30–41.

[bjhp70003-bib-0057] Nuffield Trust . (2024). Care and support for long term conditions. https://www.nuffieldtrust.org.uk/resource/care‐and‐support‐for‐long‐term‐conditions#:~:text=Background,with%20medication%20or%20other%20therapies.

[bjhp70003-bib-0058] Paterson, B. L. (2001). The shifting perspectives model of chronic illness. Journal of Nursing Scholarship, 33(1), 21–26. 11253576 10.1111/j.1547-5069.2001.00021.x

[bjhp70003-bib-0059] Pietkiewicz, I. , & Smith, J. A. (2014). A practical guide to using interpretative phenomenological analysis in qualitative research psychology. Psychological Journal, 20(1), 7–14.

[bjhp70003-bib-0060] Popov, J. , Farbod, Y. , Chauhan, U. , Kalantar, M. , Hill, L. , Armstrong, D. , Halder, S. , Marshall, J. K. , Moayyedi, P. , & Kaasalainen, S. (2021). Patients' experiences and challenges in living with inflammatory bowel disease: A qualitative approach. Clinical and Experimental Gastroenterology, 14, 123–131. 33953591 10.2147/CEG.S303688 PMC8088978

[bjhp70003-bib-0061] Pothemont, K. , Quinton, S. , Jayoushe, M. , Jedel, S. , Bedell, A. , Hanauer, S. B. , Mutlu, E. A. , & Taft, T. H. (2021). Patient perspectives on medical trauma related to inflammatory bowel disease. Journal of Clinical Psychology in Medical Settings, 29, 459–679. 10.1007/s10880-021-09805-0 34292456

[bjhp70003-bib-0062] PURC‐Stephenson, R. (2014). The posttraumatic growth inventory: Factor structure and invariance among persons with chronic diseases. Rehabilitation Psychology, 59(1), 10. 24446672 10.1037/a0035353

[bjhp70003-bib-0063] PURC‐Stephenson, R. , Bowlby, D. , & Qaqish, S. (2015). “A gift wrapped in barbed wire” positive and negative life changes after being diagnosed with inflammatory bowel disease. Quality of Life Research, 24(5), 1197–1205. 25359590 10.1007/s11136-014-0843-0

[bjhp70003-bib-0064] Rogers, C. (1951). Client centred therapy. Constable and Company Ltd.

[bjhp70003-bib-0065] Rzeszutek, M. , Oniszczenko, W. , & Gruszczyńska, E. (2019). Satisfaction with life, big‐five personality traits and posttraumatic growth among people living with HIV. Journal of Happiness Studies, 20(1), 35–50.

[bjhp70003-bib-0066] Sajadinejad, M. S. , Asgari, K. , Molavi, H. , Kalantari, M. , & Adibi, P. (2012). Psychological issues in inflammatory bowel disease: An overview. Gastroenterology Research and Practice, 2012(2012), 106502. 22778720 10.1155/2012/106502 PMC3388477

[bjhp70003-bib-0067] Severs, M. , Spekhorst, L. M. , Mangen, M.‐J. J. , Dijkstra, G. , Löwenberg, M. , Hoentjen, F. , Devan, D. , Meulen‐Jong, A. E. , Pierik, M. , Ponsioen, C. Y. , & Bouma, G. (2018). Sex‐related differences in patients with inflammatory bowel disease: Results of 2 prospective cohort studies. Inflammatory Bowel Diseases, 24(6), 1298–1306. 29688413 10.1093/ibd/izy004

[bjhp70003-bib-0068] Smith, J. , Flowers, P. , & Larkin, M. (2009). Interpretative phenomenological analysis: Theory, Method and Research. Sage.

[bjhp70003-bib-0069] Smith, J. A. (2011). Evaluating the contribution of interpretative phenomenological analysis. Health Psychology Review, 5(1), 9–27.

[bjhp70003-bib-0070] Smith, J. A. , & Nizza, I. E. (2022). Essentials of interpretative phenomenological analysis. American Psychological Association.

[bjhp70003-bib-0071] Smith, J. A. , & Osborn, M. (2003). Interpretative Phenomenological Analysis. In J. Smith (Ed.), Qualitative psychology: A practical guide to research methods. Sage.

[bjhp70003-bib-0072] Sperber, A. D. , Bangdiwala, S. I. , Drossman, D. A. , Ghoshal, U. C. , Simren, M. , Tack, J. , Whitehead, W. E. , Dumitrascu, D. L. , Fang, X. , & Fukudo, S. (2021). Worldwide prevalence and burden of functional gastrointestinal disorders, results of Rome foundation global study. Gastroenterology, 160(1), 99–114. 32294476 10.1053/j.gastro.2020.04.014

[bjhp70003-bib-0073] Springer, F. , Friedrich, M. , Kuba, K. , Ernst, J. , Glaesmer, H. , Platzbecker, U. , Vucinic, V. , Heyne, S. , MEHNERT‐Theuerkauf, A. , & Esser, P. (2023). New progress in an old debate? Applying the DSM‐5 criteria to assess traumatic events and stressor‐related disorders in cancer survivors. Psycho‐Oncology, 32(10), 1616–1624. 37695318 10.1002/pon.6213

[bjhp70003-bib-0074] Sun, Y. , Li, L. , Xie, R. , Wang, B. , Jiang, K. , & Cao, H. (2019). Stress triggers flare of inflammatory bowel disease in children and adults. Frontiers in Pediatrics, 7, 432. 31709203 10.3389/fped.2019.00432 PMC6821654

[bjhp70003-bib-0075] Taft, T. H. , Quinton, S. , Jedel, S. , Simons, M. , Mutlu, E. A. , & Hanauer, S. B. (2021). Posttraumatic stress in patients with inflammatory bowel disease: Prevalence and relationships to patient‐reported outcomes. Inflammatory Bowel Diseases, 28, 639. 10.1093/ibd/izab152 PMC8344426 34137449

[bjhp70003-bib-0076] Tedeschi, R. G. , & Calhoun, L. G. (1996). The posttraumatic growth inventory: Measuring the positive legacy of trauma. Journal of Traumatic Stress, 9(3), 455–471. 8827649 10.1007/BF02103658

[bjhp70003-bib-0077] Trachter, A. B. , Rogers, A. I. , & Leiblum, S. R. (2002). Inflammatory bowel disease in women: Impact on relationship and sexual health. Inflammatory Bowel Diseases, 8(6), 413–421. 12454617 10.1097/00054725-200211000-00006

[bjhp70003-bib-0078] Wang, R. , Li, Z. , Liu, S. , & Zhang, D. (2023). Global, regional, and national burden of 10 digestive diseases in 204 countries and territories from 1990 to 2019. Frontiers in Public Health, 11(1), 061453. 10.3389/fpubh.2023.1061453 PMC10088561 37056655

[bjhp70003-bib-0079] Williams, W. I. (2006). Complex trauma: Approaches to theory and treatment. Journal of Loss and Trauma, 11(4), 321–335.

[bjhp70003-bib-0081] Yeshi, K. , Ruscher, R. , Hunter, L. , Daly, N. L. , Loukas, A. , & Wangchuk, P. (2020). Revisiting inflammatory bowel disease: Pathology, treatments, challenges and emerging therapeutics including drug leads from natural products. Journal of Clinical Medicine, 9(5), 1273. 32354192 10.3390/jcm9051273 PMC7288008

